# The perception of public transport drivers (PTDs) on preventing road traffic injury (RTIs) in Vanuatu: a qualitative study

**DOI:** 10.1080/17482631.2022.2047253

**Published:** 2022-03-06

**Authors:** Saen Fanai, Masoud Mohammadnezhad

**Affiliations:** a Department of Public Health, Ministry of Health, Port Vila, Vanuatu; b School of Public Health and Primary Care, Fiji National University, Suva, Fiji

**Keywords:** Public transport drivers, road traffic injuries, risk factors, prevention, Vanuatu

## Abstract

**Purpose:**

Road Traffic Injury (RTI) is major public health concern globally and is excessively affecting vulnerable road users in the pacific Island nations. This study aimed to explore and understand the perception of Public Transport Drivers (PTDs) on risk factors and the existing prevention strategy of RTI in Vanuatu.

**Methods:**

This study employed qualitative methods that used 31 In-Depth Interviews (IDIs) to gather data from PTDs from 14 October to 30 November 2020. Purposive sampling was used to recruit PTDs from three main municipalities, Luganville, Port Vila and Lenakel. Semi-structured open-ended questionnaire were used to gather data. Interview data was transcribed and manual thematic analysis was performed to analysis the data.

**Results:**

Data saturation was reach from interviewing 31 PTDs who were all male. Five main themes were generated from the study including the trend of RTI, the determinants of RTI, high-risk road users, traffic law enforcement and public education. The respondents perceived that the best measures for preventing RTI include community education, enforcement of road traffic control laws and addressing specific road infrastructure issues.

**Conclusions:**

Changing driver behaviours, community education on road safety and enforcement of traffic laws are essential for preventing RTI in Vanuatu.

## Introduction

Road Traffic Injury (RTI) is major public health concern globally, excessively affecting vulnerable road users, including pedestrians and riders in Low and Middle Income Countries (LMICs; Organization WH, 2018). Existing trends suggest that this will continue to be the case in the future. Reports indicate that only 1% of the world’s motor vehicles are in LMICs but 13% of deaths occur in these countries(Herman, Ameratunga, Jackson et al., 2012a; Nakahara et al., 2013). Approximately 1.35 million people die each year as a result of RTIs and is the leading killer of people aged between 5 and 29 years (Cameron, 2004; Organization WH, 2018). Globally, RTI is among the top 10 leading cause of death for people of all ages. The risk of someone dying from RTI is more than 3 times higher in LMIC than in high-income countries (Gopalakrishnan, 2012; Organization WH, 2018). While there is global improvement to control and prevent communicable diseases, Non-Communicable Diseases (NCDs) and injuries are increasing. Today RTI kills more people than tuberculosis and HIV/AIDS commonly known to be the leading causes of death globally (Nakahara et al., 2013; Organization WH, 2018; Racioppi et al., 2004).

More than half the people injured or killed in RTA are youths and adults aged between 15 and 44 years who are often the providers in a family. Injuries and disabilities caused from RTA adds to inactivity that contribute to NCDs that include ischaemic heart disease, diabetes and stroke (Addy et al., 2004; Racioppi et al., 2004). Our societies therefore endure an incredible cost that is evaluated to be about 2% of total gross domestic product in some countries (Herman, Ameratunga, Jackson et al., 2012b; Ipingbemi, 2008). In our region, it is predicted that increasing motorization will make RTI the leading cause of death among young people beyond 2020 (Organization WH, 2018; Sharma, 2008). It is evident in various parts of the world that lives could be safe and injuries prevented when road traffic laws are enforced(Mphela, 2011). If not addressed, RTI will become one of the leading causes of morbidity and mortality in Vanuatu, next to diabetes and hypertension (Herman, Ameratunga, Jackson et al., 2012a; Organization WH, 2018).

Constantly increasing, RTI is a public health concern in the Pacific Islands region including Vanuatu but it has been given little attention (Herman, Ameratunga, Jackson et al., 2012a; Nakahara et al., 2013). While a significant progress is been made in reducing mortality from RTI among high-income countries, there has been no reduction in any LMIC (Addy et al., 2004; Kumeda et al., 2019; Organization WH, 2018). Improvement made in reducing the road traffic deaths significantly varied between countries and regions of the world (Cameron, 2004). Very few studies on RTI have been conducted in the Pacific Island region (Herman, Ameratunga, Jackson et al., 2012a; Sharma, 2008). There is an urgent need to expand knowledge on RTI situations in this region. Public Transport Drivers (PTDs) are important road users who witness and deal with RTI issues at a daily basis, their views on preventing RTIs are not often observed but are of utmost important. More than half of all road traffic deaths are among vulnerable road users that include PTDs. Their views will form the basis upon which we can generate information on specific RTI preventive measures.

In Vanuatu, despite the 2007 Traffic Control Act (TCA) that limits vehicle speed to 40 km/hr. in build-up areas over speeding is the main cause of RTA and RTI (Luen, 2018; Spickett et al., 2013). The road traffic death rate in Vanuatu is 16 per 100,000 populations. This is 3 times higher than Australia and Fiji, 5.4 and 5.8 per 100,000 populations, respectively, and is much higher than in the USA 10.6 per 100,000 populations and ranks Vanuatu 91 in the world (Nakahara et al., 2013; Organization WH, 2018). This indicates the shocking reality by such a plague and demonstrates that the Vanuatu roads are of the most dangerous or that Vanuatu drivers are the most reckless in the region (Organization, 2004).

There are significant gaps in knowledge regarding RTI including the associated contextual factors in the pacific hindering the prevention efforts. Consequently, it places the vulnerable populations and their families at increased risk of being trapped in poverty. So, this qualitative study aims to explore and understand the perception of PTDs on preventing RTI in Vanuatu. It likewise offers an approach for pre-diagnosing the significant risk factors on RTI in Vanuatu, recognizes gaps in preventing RTI and decides the best way forward for development.

## Methodology

### Study design and setting

This is a qualitative study that used In-depth Interviews (IDIs) on PTDs in three municipalities of Vanuatu between 1 October and 15 November 2020. The three municipalities were the only municipalities in the country located in three out of the six provinces of Vanuatu, Lenakel municipality in Tafea Province, Port Vila Municipality in Shefa province and Luganville municipality in Sanma province. IDI provides very rich information and it offers the opportunity to ask follow-up questions, probe additional information, justify previous answers, and establish a connection between several topics. It also offers a comfortable atmosphere where people can establish conversation with calmness (Guion et al., 2011; Manzano, 2016; Queirós et al., 2017).

### Study sample

All PTD in Lenakel, Port Vila and Luganville municipalities who meet the inclusive criteria were selected to participate. The PTDs neither than service bus drivers, taxi drivers and truck transport drivers were included. These PTDs have to be 18 years old or over, have more than 6 months working experience in the land transport industry and were self-identified as a Ni-Vanuatu. PTDs who are medically unfit and those who were unwilling to participate were excluded from the study.

This study employed purposive sampling because it is a cost-effective and time-effective sampling method and was the appropriate method for this study given the small number of primary data sources who will contribute to the study (Tongco, 2007). Data saturation was reached after 31 (Pius et al.Pius et al.Pius et al.Pius et al.Pius et al.Pius et al.Pius et al.Pius et al.Pius et al.) PTD participants were interviewed.

### Data collection tool

An interview guide was developed to answer the research questions. The interview guide was developed based on relevant literature reviews paying special attention to the research questions and had two sections that included a demographic section and 5 open-ended questions. The interview guide was used to probe elicit information from participants and allowed participants to express their personal views freely (Hertlein & Ancheta, 2014). The demographic questions were aimed at collecting demographic characteristics that include participant gender, age, education level, years of experience, where they grew up and their marital status. All the questions and interviews were conducted in Bislama (Vanuatu common dialect). To ensure standardization of the interview guide, a targeted respondent approach was used where only PTDs participated, furthermore the interview guide were pretested. Pre-testing involved 5 PTDs of Port Vila municipality who were not included in the study. The interview guide was pre-tested to ascertain if the questions, directions, and language used were clear to the respondents in term of their requisites. The responses from the pre-test were used to correct flaws in the question items and re-adjustment were done to meet response expectations.

### Study procedure

Copies of the information sheet were made available to potential participants at the main transport centres of the respective municipalities. Written consent was mandatory for this study thus, those participants who agreed to participate were requested to complete and signed a consent form. Witnessed verbal consent and permission to audio-record the discussions were obtained. Confidentiality of our participants were rest assured, this so they give out information without bias. Recruitment of participants were done autonomously and IDIs were conducted by the main researcher at the public transport centres and sub centres at times convenient to the participants. The main researcher ensured that all interviews were conducted in isolation from other potential study participants who may influence responses. When conducting the interviews, questions were asked in a way starting with simple and general questions then gradually progressing to more specific ones. All IDIs begun with asking questions on demographic information and then proceeding to the semi-structured questions. Throughout the interviews probing techniques were performed according to the reflections of each participant, concerning experiences of RTIs and what is perceived about RTIs. After each interview, preliminary data analysis took place simultaneously in order to identify ideas that can guide the next interview. All interviews were conducted in Bislama. During the course of the study, the researcher maintained a maximum five IDIs per day to avoid exhaustion that consequently would affect the quality of questions asked. Depending on the responses, each interview took 20 to 25 minutes. All interviews were recorded using a voice recorder. Data collection was continued until saturation of each concept was reached and further data collection failed to contribute new information (Bowen, 2008).

### Data management and analysis

Data from digital recorders and any additional notes taken during IDIs were transcribed using Microsoft Word. Each IDI transcript was checked twice—once immediately after transcription by the researcher and research assistants and again during data analysis. The transcripts were compared with the recorded digital files for accuracy. Disagreements or issues needing further clarity were resolved through discussions and triangulation of data sources. Manual thematic analysis was performed for IDIs based on exploring both predetermined issues of interest and looking for new issues raised by the respondents (Boyatzis, 1998; Green et al., 2007). Themes representing the research topic area were listed and coded based on frequency and order of mention. Open coding was done and codes were grouped into categories, and themes identified (Graneheim & Lundman, 2004). Data analysis involved summarizing large raw data quantities, categorizing and rearranging of the data. The data collected were sorted out and tabulated in a form of tables to pave way for further analysis.

### Study rigour

A good rigorous research must be reliable and valid (Cypress, 2017). In this study, both male and female participants of different social backgrounds who were not physically retarded and were able to hear and speak fluently were given equal opportunities to participate. It is credible because it measures what was intended and was a true reflection of the social reality of the participants, reflected by the study participants who had engaged in RTI issues for prolonged period. Additionally, the findings could be transferred to other contexts or settings particularly in the Pacific Island region as the participants are not unique to other regional or LMIC settings. Moreover, the study outcome can be relied on because the development process was described in sufficient detail to facilitate another researcher to repeat the work.

### Ethical considerations

This study was approved by Fiji National University’s (FNU) College Health Research & Ethics Committee (CHREC) and the Vanuatu Ministry of Health research and ethics committee. Study participants were informed that their participation was voluntary, and information gathered in the interviews and discussions would be confidential. Information explaining the aim of the study were provided orally and in writing before a written consent were sought from respondents.

## Results

### Demographic characteristics

Demographic characteristics of PTDs who participated in this study are summarized in Table I below. Thirty-one PTDs including mini bus drivers, taxi drivers and transport truck drivers participated. Entirely the participants agreed to participate. All the respondents (100%) were male and their age groups almost equally distribute with the majority (52%) of them being over 40 years old. Their level of education varies with the majority of them (61%) only attended up to primary school level. According to their years of employment, 48% of them have been working between 11 and 20 years. It is also noted that 52% of the participants grew up in town. Moreover, the majority of them are married (81%) as compared to those that are single.

**Table I.** Profile of PTDs (n = 31)

Respondent	Categories	Frequency(n)	Percentage (%)
Gender	Male	31	100
	Female	0	0
Age	25 − 40 years old	15	48
	41–60 years old	16	52
Level of Education	≥ Tertiary	1	4
	High School	9	29
	Primary School	19	61
	No school	2	6
Number of years employed	0–10 years	9	29
	11–20 years	15	48
	≥ 21 years	7	23
Grew up	In town	16	52
	In the Village	15	48
Marital status	Married	25	81
	Single	6	19

### Perception of PTDs

The study identified several major themes, including the trend of RTA/RTI, the determinants of RTA, high-risk road users, traffic law enforcement and public education. Fifteen sub-themes emerged from these five main themes (Table II). Further thematic analysis of PTDs interviews are described below under each main themes and sub themes including the samples of interview responses quoted. Each interview sample quoted are labelled with the age and gender of the respondent including the unique participant identification number PTD 1 and so on.

**Table II.** Main themes and sub-themes for PTDs

Themes	Sub-themes
The trend of RTA/RTI	The extend of RTI
	The severity of RTA and RTI
The determinants of RTA	Irresponsible driver behaviour
	Road infrastructure
	Vehicle conditions
High-risk road users	Young drivers
	Passenger seeking attitude
	Pedestrian ignorance of traffic
Traffic law enforcement	Ineffective enforcement
	Penalties for traffic law offenders
	Corruption in traffic law enforcement
	Poor compliance to traffic law
Public Education	Lack of awareness on traffic laws
	Lack of first aid knowledge to respond to road accident trauma
	Lack of general knowledge to report RTA and RTI

#### Theme 1: the trend of RTA/RTI

When asked about RTI situation in Vanuatu, two subthemes that include the extend of RTI and the severity of RTI emerged

### The extend of RTI

Almost all the respondents perceived that in the last five years, there has been increasing trends of RTA and RTI and the case fatality rates have increased shockingly. One of the respondent stated,

Over the last five years RTA have become seriously worse than ever before. There is almost news of road traffic accident everyday including injuries or deaths. (40 years old male, PTD7)

RTI occurs at a daily basis, another respondent stated,

It is becoming scary as we have almost at a daily basis give way to ambulances that later we learn there is RTA and people are injured”. (30 years old male, PTD13)

The roads are no longer safe, one respondent stated,

Our roads are no longer safe; there is RTA every day and more people are getting injured and losing lives. (43 years old male, PTD10)

### The severity of RTA and RTI

The respondents perceived that injuries from road traffic crashes today a far worse than it was more than five to ten years ago and the injuries are devastating. One of the respondent stated,

Many accidents today result in very bad injuries and in most cases the vehicles are written-off. (50 years old male, PTD23)

There is observed increase mortality from RTI, one other respondent stated,

In the past we do not often hear people die in RTA, however in the last five years many people especially the young people have lost their lives in RTA. (42 years old male, PTD24)

There is increase hospitalization from RTI, one respondent claimed,

Today when a RTA occur someone must die or have a live threatening injury. If you go to our hospitals, they are full of RTI people. (48 years old male, P29)

#### Theme 2: the determinants of RTA

Three subthemes including irresponsible driver behaviour, road infrastructure and vehicle condition emerged when asked about what they perceived to be the causes of these RTAs and RTIs.

### Irresponsible driver behaviour

According to the interviewees when drivers or passengers do not follow traffic laws, they put themselves and others at risk of accidents that can cause serious injuries and even death. One of the respondent stated,

One of the major causes of RTA is driving under the influence of alcohol or drugs; many drivers choose to ignore the traffic law that do not drink when driving. (48 years old male, PTD29)

It is observed that drivers use mobile phones when driving, one other respondent stated,

Many drivers want talk on the phone when driving and that is very risky. And the risk increases with speeding. (30 years old male, PTD18)

There is observed relationship between tiredness and RTA one responded claimed,

Some accident occurs because the driver is tired either after attending a major event (party) or after a long day’s work without having enough rest. (28 years old male, PTD15)

### Road infrastructure

The respondents overwhelmingly described poor road infrastructures include those where a road defect directly triggers a crash as major contributing factors. The respondents raise concerns on poor road conditions, traffic volume and road traffic aids. One respondent stated,

Our roads are not safe during rainy seasons because the roads have too many pot-holes. Furthermore, the poor road drainage system results in road flooding that has contributed to a lot of accidents. (35 years old male, PTD1)

The absence of road traffic signs is an issue, one respondent stated,

Our roads do not have traffic signs, existing traffic signs are old, faded, vandalized, non-reflective and obstructed by vegetation, new drivers often got involved in RTA on these roads (35 years old male, PTD3).

Increase traffic volume is becoming worrying because the roads remain same, one respondent claimed,

There are no new roads and these roads are getting smaller due to increase traffic volume. We have plenty accidents because the number of vehicles have increased excessively but the roads have not changed. (49 years old male, PTD11)

### Vehicle conditions

Additionally, the respondents perceived that vehicle defects have contributed to major RTA on our roads. One of the respondent stated,

Most accidents on the road involved defect vehicles, the brakes fail, the wheels burst or the engine just suddenly stops because the vehicle is old or not properly maintained. (46 years old male, PTD13)

One other respondent claim corruption is also an issue,

Poor condition vehicles that secure road worthiness certificate through corruption ends up involving in very bad accidents. (48 years old male, PTD19)

Another respondent stressed on the issue of vehicle appearance vs the working condition,

Many of the reconditioned vehicles like the services buses look new on the outside but the engines and other parts are not working well, many accidents involve them. (44 years old male, PTD27)

#### Theme 3: high-risk road users

Three sub themes arose from the list of high-risk road users that were described and they include being a young driver, passenger seeking attitude and individual who are ignorant of traffics.

### Young drivers

According to the respondents the risk of RTA increases when the vehicle driver is too young. One of the respondents stated,

The majority of RTA that occur involves young drivers; they lack the experience and the confidence to drive well. (50 years old male, PTD9)

Young drivers are easily distracted, another respondent stated,

Young drivers are easily distracted and thus always involve in RTA. (49 years old male, PTD11)

Young drivers lack the driving experiences, one respondent stressed,

Many young drivers never had a good experience driving before being in charge of public transports, we can always question how they got their driver’s license and public transport permits at the first place. (48 years old male, PTD29)

### Passenger seeking attitude

RTA occur when drivers decide stop the vehicle suddenly anywhere and anytime they want. One of the respondent stated,

With increasing demand for public transports to make money there are often issues with drivers who stop their vehicle anytime and anywhere for passengers that results in vehicle clash (36 years old male, PTD17)

Another respondent described,

Some public transport drivers think they own the roads and they stop anywhere they want to pick or drop off passengers and it is becoming risky for these passengers and other vehicles. (50 years old male, PTD23)

Some driver attitudes are becoming dangerous, one respondent claimed,

The public transport driver attitudes to stop anywhere for passengers have seen several accidents occur and passengers got injured. (42 years old male, PTD7)

### Pedestrian’s ignorance of traffics

Pedestrians who take for granted that drivers are looking and can stop the vehicle any time are at increased risk of involving in RTA and RTI. One respondent stated,

Many pedestrians become victims of RTA because they are careless when walking on the road, they just do not care of incoming vehicles. (38 years old male, PTD31)

Drivers cannot be always blamed, one other respondent stated,

We cannot blame drivers for all the accidents, many pedestrians take it for granted that drivers are cautious but end up injured because drivers only look straight and only occasionally to the sides. Anyone can be hit anytime coming from the side. (36 years old male, PDT20)

Pedestrians are careless, one respondent stressed,

Some pedestrians cross the roads anytime they want and expect oncoming vehicles to give way; this is very risky as drivers who are not concentrating may hit them. (47 years old male, PTD28)

#### Theme 4: traffic law enforcement

Four subthemes that include ineffective enforcement, corruption in law enforcement, penalties for traffic law offenders and driver compliance to traffic rules emerged when asked about the challenges faced.

### Ineffective enforcement

According to the respondents, the intervention of traffic law enforcement programs in the country is not working and have resulted in the increase of RTA over the last 5 years. One of the responded stated,

Enforcement officers are not physically present on our roads that’s why you see many crazy drivers driving around town. (63 years old male, PTD4)

The presence of TLO are becoming irregular, one other respondent stated,

Our enforcement officers are only seen on the road during special operations example after the dateline to renew permits passed, but every other time you do not see them. (56 years old male, PTD6)

There is increase traffic law offenders when enforcement is weak, one respondent also stated,

When you keep seeing vehicles with drunk drivers on the road that is an indication that there is no road traffic law enforcement. (24 years old male, PTD5)

Weak enforcement allows for offenders to remain on the roads, one respondent expressed,

It is funny when we see drivers who have recently been booked for causing a car crash because of driving under the influence of alcohol still driving around. (46 years old male, PTD16)

### Penalties for traffic law offenders

The respondents claim that penalties are imposed on traffic law offenders to punish the offender and to deter any future criminal acts, but it has not work for Vanuatu. One of the respondents stated,

Penalties for breaking traffic rules here is not severe enough to discourage reoffending. A fine of 5,000VT is nothing to most public transport drivers, they can pay up and remain careless as reoffending will only cost 5,000VT. (47 years old male, PTD28)

There is need to increase penalties for road traffic law offenders, one other respondent stated,

The fines for breaking traffic laws is too low, they should increase the amount so that people are mindful when they drive on the roads. (63 years old male, PTD4)

Traffic law offenders should end up in jail, one other respondent stressed,

When they start sending traffic law offenders to prison that is when we can see change to our road accident situation. Harsher penalties should be serve for reoffenders like removing their driver’s license for 5 years or so (47 years old male, PTD22)

### Corruption in traffic law enforcement

It was also observed through the interviews that corruption in the traffic police in the form of bribery and extortion was an issue. One respondent stated,

Some of our enforcement officers are corrupt; you can just pay your way out of a bad situation. If you are able to pay a good money to a traffic officer, you can be assured your case close. That is why reoffenders are seen on our roads. (63 years old male, PTD4)

Corruptly intimidation is common, one other respondent stated,

Sometimes when you are got, enforcement officers may intimidate you like saying do you want to go to jail? So offenders fall to their threat and pay them so much money in fear to avoid jail or heavy fines. (28 years old male, PTD15)

Corruption allows for increase in the number of young drivers, one respondent stated,

The increase in the number of RTA is related to the increase in the number of young drivers because of corruption. Traffic officers issue driver’s license in corrupt manner, people don’t follow proper process and procedures to qualify for one, example you can buy a driver’s license without getting a learner’s license. (49 years old male, PTD11)

### Poor compliance to traffic laws

The respondents perceived that when enforcement is weak, general non-compliance to road traffic law on the other hand rises. One of the respondents stated,

I noticed that today people no longer respect our traffic rules, they can speed, they can stop and they can make U turns anywhere they want. This has given rise to road accidents. (56 years old male, PTD6)

Basic traffic rules are not followed, one other respondent stated,

Many new drivers do not follow basic traffic rules; it is either they choose to ignore them or they do not know the rules because they did not follow proper process to get their driver’s license. (48 years old male, PTD29)

One respondent stated,

Driving on our roads is very dangerous because some drivers do not follow traffic rules, they can stop all over sudden or they can turn anywhere without switching their indicator light. It is becoming worse. (36 years old male, PTD17)

#### Theme 5: public education

When asked about their perception on the contributing factors to RTI three subthemes that include the lack of awareness on traffic laws, the lack of knowledge to respond to road accident trauma and the lack of general knowledge to report RTI emerged.

### Lack of awareness on traffic laws

According to the respondents, while it is expected of drivers to know the traffic laws, the general public should be equally informed of these traffic rules. One of the respondent stated,

The majority of people using the roads do not know at least the basic laws of traffic. Most of the time we blame the drivers but I think many times it is also the fault of passengers or pedestrians. (37 years old male, PTD27)

One other respondent added,

We expect traffic law officers to come out more on TV, radio or other media outlets and inform the public on road safety, this is rarely happening. (33 years old male, PTD14)

Another respondent stated,

I don’t see it happening but I think the authorities can use schools to make educate the population at large on safety road measures. The kids will extend the knowledge to the community when they return home. (48 years old male, PTD29)

### Lack of first aid knowledge to respond to road accident trauma

It was significant from the interviews that many drivers and pedestrians do not have the basic first aid knowledge to care for road injury victims or at least know what to do when they are faced with one. One of the respondent’s stated,

To be honest, many of us don’t know what to do than to try to pull the victim off the crashed vehicle when we come across one. All we can do on the spot is to try to call the ambulance, it is often very scary. (32 years old male, PTD12)

There is need for first aid training for drivers, one other respondent added,

As drivers we need to know how to apply first aid in RTA, most of the time we witness the scene or be the first to attend to one. The authorities should organize trainings or awareness on first aid for public transport drivers. (63 years old male, PTD4)

Drivers could be of help had they know what to do, one respondent stated,

Many times we join the by standers and cause overcrowding at the scene. Had we know what to do, we could be of some assistance to the police or emergency response team. (43 years old male, PTD10)

### Lack of general knowledge to report RTA and RTI

It was significantly highlighted that many people do not know who and how to contact the appropriate response team for RTA and RTI. One of the respondent stated,

It is easier to call pro-medical because 112 is an easy number to remember. We do not know the police and hospital emergency numbers. It keeps changing. (40 years old male, PTD8)

There is luck of general knowledge to communicate appropriate information on RTI, one other respondent stated,

There should be awareness because we do not know emergency numbers, who to call, when to call and what to report when we are at the scene of the accident. Sometimes we call our relatives in the workforce and they communicate the information for us or they give us the appropriate contact details, this is too much time wasted. (42 years old male, PTD2)

There is unnecessary delay due to lack of understanding, one respondent stated,

Sometimes people call the police only or the ambulance only to inform of the accident, but both of them may be required on the scene. Too often they call the police only and then upon arrival the police will call the ambulance. Sometimes the victim dies even before reaching the hospital. (30 years old male, PTD13)

## Discussion

This study explored the perception of PTDs on preventing RTIs in Vanuatu, the findings based on the results were summarized under five relevant major themes that include the trend and determinants of RTI, high-risk road users, traffic law enforcement and public education.

The determinants of RTI

The findings indicate that irresponsible driver behaviour, road infrastructure and vehicle conditions are key risk factors for RTIs in Vanuatu.

### Irresponsible driver behaviour

Irresponsible driver behaviour characterized by driving when fatigue, driving when under the influence of psychoactive substances, use of mobile phone while driving and speeding as factors strongly associated with RTI.

The major causes of RTAs and related injuries in Vanuatu are associated with driving under the influence of alcohol. The weak traffic law enforcement interventions could be linked to this irresponsible behaviour. Alcohol remains one of the main contributing factors of traffic accidents (Sutlovic et al., 2014). Driving After Drinking (DAD) appears to be a fairly common behaviour among youths. The common risk factors associated with DAD are sex, fraternity or sorority affiliation, family history of alcohol abuse status and alcohol consumption relating to both personal attitudes and alcohol expectancies for sexuality. Consistent with prior research, males, medium or heavy drinkers (as compared to light drinkers), and participants with stronger approval of drinking-driving were most likely to engage in DAD (LaBrie et al., 2011). Moreover, drivers become tired after driving long hours but force themselves to drive ignoring the symptoms of fatigue including sleepiness and trouble focusing. Fatigue characterized by Sleepiness hinders the driver’s ability to focus properly on the road thus often resulting in RTAs and injuries. Driver fatigue has detrimental effects on driving performance and the effects differ under different roadway geometries. Fatigue impaired drivers’ abilities to control the steering wheel, and the impairment proved more obvious on curves (Du et al., 2015).

Equally so, the use of mobile phones when driving is a contributing factor to RTI. The number of RTIs started to rise steadily after the year 2010 that was several years after mobile phones were first introduced in Vanuatu. Nevertheless, recent RTAs and associated injuries in Vanuatu have been linked to the use of mobile phones when driving. Mobile phone use while driving is a major public health problem highlighted by high rate of mobile phone use among individuals associated with crashes, at 73% (Bener et al., 2006). Using mobile phones can cause drivers to take their eyes off the road, their hands off the steering wheel, and their minds off the road and the surrounding situation. Evidence shows that the distraction caused by mobile phones can impair driving performance in a number of ways, e.g., longer reaction times (notably braking reaction time, but also reaction to traffic signals), impaired ability to keep in the correct lane, and shorter following distances. Text messaging also results in considerably reduced driving performance, with young drivers at particular risk of the effects of distraction resulting from this use (Organization WH, 2011).

The findings also point to speeding as an irresponsible driver behaviour that poses an increased risk to RTI. Speeding associated with other risk factors like being a young driver and driving under the influence of alcohol increases the likelihood of RTA with severe RTI. Vanuatu has one of the high rates of RTI involving young drivers. Most victims of RTI who present to hospital emergency units are intoxicated. Young and intoxicated drivers like to speed. Drivers easily lose control of vehicle when they speed thus resulting in RTI. Speeding contributes to the increased risk of losing vehicle control. At higher speeds, cars become more difficult to manoeuvre—especially on corners or curves or where evasive action is necessary (Pius et al.Pius et al.Pius et al.Pius et al.Pius et al.Pius et al.Pius et al.Pius et al.Pius et al.). Studies of the relationship between the survival of a vulnerable road user and vehicle impact speed (the speed at which the vehicle was travelling when it hit the vulnerable road user) show that small increases in travel speed can result in large increases in braking distances and impact speed—substantially increasing the risk of a pedestrian, motorcycle, bicycle rider, or baby in a pram being killed or seriously injured (Davis, 2001).

### Road infrastructure

The results point to three road infrastructure issues that include poor drainage system, inadequate roads and insufficient road traffic signs contributing factors to RTIs in Vanuatu.

Poor drainage system associating with road and pothole flooding is connected to many RTA and injuries in recent times. Vanuatu is known for its vulnerability to disastrous weather patterns including cyclones and long rainy seasons. With the poor road drainage systems, it is without doubt that road flooding is inevitable and thus RTA and injuries. During extreme weather events transport infrastructure can be directly or indirectly damaged, posing a threat to human safety, and causing significant disruption and associated economic and social impacts (Pregnolato et al., 2017).

Vehicle congestion due to increase volume of vehicle in town centres is associated with increased RTI. Vanuatu towns are known to have very narrow roads; thus, the increase in the number of vehicles often results in traffic congestion. Traffic congestions leads to careless driving in an attempt to get through by manoeuvring and speeding that increases the risk of RTAs and injuries. Traffic congestion overall has a negative impact on road safety. This may be partially due to higher speed variance among vehicles within and between lanes and erratic driving behaviour in the presence of congestion (Wang, 2010).

Moreover, the deficiency of road traffic signs and pavement markings on our main roads have contributed to many RTA and related injuries. Most Vanuatu roads and key junctions do not have road signs and pavement markings. Respondents described road traffic signs as old, faded, vandalized, non-reflective and are obstructed by vegetation. Meanwhile, the road traffic control act on road signs and signals provides that signs shall be so placed that the drivers for whom they are intended can recognize them easily and in time (Macmillan, 2016). The failure of government to provide and maintain traffic signs in order to guide road users could be associated with numerous accident black spots on the highways and other major road ways (Ezeibe et al., 2019). Traffic sign deficit has both immediate and remote implication. The immediate implication is RTAs. Traffic sign deficit is a major cause of RTAs which mostly occur at sharp bends and road intersections which are not preceded by effective traffic signs (Adedeji et al 2016).

### Vehicle condition

The findings also indicate that vehicle conditions contribute to RTI in Vanuatu and the findings point to the import and sale of recondition vehicles, use of defect vehicles and corruption in issuing vehicle road worthiness certificates as factors associate with the increased risk of RTI. Vanuatu is among the few pacific Island countries that rely on recondition vehicles from Korea and Japan because they are cheap. When you purchase a new vehicle, you expect it to be free from defects, this could not be the case with recondition vehicles. The recent increase in the import and sale of recondition vehicle in the local market is directly linked with the increase in the number of RTA and therefore RTI. Moreover, the increase circulation of defect vehicles on the roads is a major risk factor for RTI in Vanuatu. Defect vehicles involve problems with ignition switches, accelerators that stick or break, electrical system problems that cause fires or loss of lights, wheels that crack or break, fail steering system or brake failure that results in loss of vehicle control (Khorasani-Zavareh et al., 2013). Furthermore, corruption in the process of issuing vehicle road worthiness certificates indirectly pose the risk for RTI as it allows for defect vehicles to be on the road. Given the high rate of RTA linking to defect vehicles raises the question of how it ended up on the roads at the first place. The only legitimate reason could be corruption in the process of issuing vehicle road worthiness certificates by respective authorities. Tyres and brakes are the two most dominant components that contribute to the mechanical defects causing accidents (Van Schoor et al., 2001). Other major defect vehicle causal factor associated with motorcycle injuries include missing indicators, poor motorcycle maintenance, defective speedometers, lack of head lamps and inappropriate motorcycle chain (Pius et al.Pius et al.Pius et al.Pius et al.Pius et al.Pius et al.Pius et al.Pius et al.Pius et al.).

#### High-risk road users

The results indicate that high-risk road users include young drivers, drivers with passenger seeking behaviour and pedestrians who ignore traffic.

Young drivers are easily distracted, they do not have good driving experience and that they are not confident thus they easily lose control of vehicles when unexpected distractions occur. Young drivers aged between 18 and 25 make up a third of public transport drivers in Vanuatu according to Palgrave (Macmillan, 2016). The factors that were typically and most frequently associated with collisions ofyoung drivers include drugs or alcohol and excessive speed. Inexperience and driver distraction are link to both young driver scenarios and actual collisions (Rolison et al., 2018). Furthermore, driver passenger seeking behaviour characterized by failure to indicate stopping and taking off, failure to give way, inappropriate take overs and speeding poses major risk for RTI. In Vanuatu, the number of public transport vehicle including mini buses, taxis and pick up transports are proportionately very high compare to demand for services, this results in the emergence of these risky behaviours. The results also indicate that pedestrians with traffic ignorance behaviours are at increased risk of RTI. Pedestrians take it for granted that all drivers will give way when they step onto the pavement. This behaviour is increasing in Vanuatu where there are no traffic lights, no pedestrian crossings and drivers make their own decisions to give way for pedestrians to cross roads. People who ignore traffics had previous unpleasant experience of vehicle-collision thus developed self-protective behaviours in road-crossing (Hashemiparast et al., 2020).

#### Inefficiency in traffic law enforcement

Traffic law enforcement inefficiencies characterized by no physical presence of traffic law enforcement officers on the roads and corruption in traffic law enforcement are strongly associated with the increase risk of RTIs. In areas where there is no physical presence of traffic law enforcement officers the vehicle drivers, riders and pedestrians fail to observe basic road traffic safety rules. Institutional incapacity in terms of resources affects the ability to enforce the road traffic laws. In Botswana the enforcement of new road laws has achieved little in the reduction of fatalities. Increasing the minimum driver licencing age may be a panacea to road accidents (Mphela, 2011). Furthermore, corruption in traffic law enforcement is associated with the increase in RTIs. Bribes are often demanded in situations where road users have committed an offence such as speeding, overloading, or driving unlicensed or un-roadworthy vehicles. Bribery in these instances may be used to ensure that the offender escapes a stiffer penalty, this allows for the presence of these risky traffic activities on the roads thus increasing the risk of RTI. The most commonly paid bribe is for driver testing and licencing irregularities, landing more young incompetent drivers on the roads making the roads unsafe. Weak and corrupt enforcement is a major setback to achieving safer roads in LMICs (Okonkwo et al., 2010).

#### Public education

Road users including vehicle drivers, riders and pedestrians have inadequate knowledge required for road safety. Moreover, the general population lack the information on RTI situation and how to prevent RTI. The lack of awareness on TV, Radio and newspapers is evident to the lack of information on the issue. Community and school education programs on road safety is fundamental for preventing RTI. Pedestrian safety education can change observed road crossing behaviour (Duperrex et al., 2002). Campaigns combined with increased police enforcement appear to be more effective than campaigns without (Hoekstra & Wegman, 2011). In contrast, a study conducted in Kenya by Walter A et al. on the impact of road safety awareness campaign on motor cycle-related road traffic injuries showed that the compliance with road safety measures among motor cycle riders remain low after road safety after mass awareness campaign intervention (Odhiambo et al., 2016). There is no reliable evidence supporting the effectiveness of pedestrian education for preventing injuries in children and inconsistent evidence that it might improve their behaviour, attitudes, and knowledge (Ellison et al., 2015). Further review of similar literature is necessary to draw further conclusions.

#### Study strengths and limitations

This study is the first of its kind to be conducted in Vanuatu and in the Pacific region. Being qualitative in nature this study gained a deeper understanding of what people perceived around RTA in Vanuatu, rather than a surface description of a large sample of a population. It is credible because it measures what was intended and was a true reflection of the social reality of the participants who are engaged with RTA and RTI matters for prolonged period. The findings in this study could be transferred to other contexts or settings particularly in the Pacific Island region as the participants are not unique in this region and other LMIC settings. The findings in this study can form the basis for new policy development as well as further studies in related topics.

In this interview-based study on RTA prevention, opinions were collected only from land transport taxi and bus drivers. The study had a relatively small number of participants, but all participants were experienced and knowledgeable. Saturation was reached for all ideas among the participants.

## Conclusion

This study helps us understand issues around RTI, it highlights important risk factors of RTI in Vanuatu that if considered and addressed appropriately, good road safety policies and strategies could be developed and then prevention of RTIs will become attainable. RTI prevention initiatives are hampered by the ineffective communication of information on the issue across all level of society therefore the need for community education on road traffic safety. RTI can be prevented by changing driver behaviour, community education on road safety and enforcement of road traffic control laws.
